# Why men are the minority: The perceptions of young men in UK post-primary education about studying psychology at university

**DOI:** 10.1371/journal.pone.0322541

**Published:** 2025-05-08

**Authors:** Elida Cena, Stephanie Burns, Ruth Lee, Kathryn Gillespie, Patrick A. O’Connor, Tara Anderson, Grace Duffy, Lisa Graham-Wisener

**Affiliations:** 1 School of Psychology, Queen’s University Belfast, Belfast, United Kingdom; 2 School of Education, Language and Psychology, York St. John University, York, United Kingdom,; 3 School of Nursing & Midwifery, Queen’s University Belfast, Belfast, United Kingdom; Lamar University, UNITED STATES OF AMERICA

## Abstract

Psychology is categorised as a science by most higher education authorities internationally. As with many science, mathematics and engineering fields, women are underrepresented in psychology at senior levels (the ‘leaky pipeline’). However, it is men who are underrepresented within the discipline overall, meaning that the psychology workforce does not reflect the population it aims to serve. It is important to understand why fewer men are opting to enter the profession, beginning with their choices regarding higher education. The current study is one of the first to qualitatively examine the perceptions and motivations of adolescent and young adult men in post-primary education of the study of psychology at university undergraduate level. Twelve focus groups were conducted with young men (*n *= 64) studying AS or A2-Levels, between 16 and 18 years of age, in post-primary schools in a UK region, Northern Ireland (NI). Thematic analysis demonstrated that psychology was viewed in a gendered way as a ‘feminine’, ‘soft’ subject dealing with emotions, and was not perceived as an ‘objective’, ‘fact-based’ science. The lack of male role models in psychology was a barrier to young men pursuing psychology at university level. Young men, whether currently studying psychology at school or not, expressed gendered career expectations and motivations. There are opportunities for targeted efforts with young men to promote psychology as a science and a multi-faceted discipline. Findings will inform the efforts of those in post-primary and higher education level in making psychology inclusive in terms of gender and improving the diversity of psychology as a field.

## Introduction

Most, if not almost all, domains of psychology are characterised by a scientific commitment to comprehensively submit hypotheses to the possibility of falsification [1]. Psychology is categorised as a science by many higher education authorities worldwide, including the UK’s Higher Education Statistics Agency [2]. While a great deal of work has set out to explain the underrepresentation of women in science, mathematics, and engineering (STEM) fields [e.g., 3,4], it is men who overall are underrepresented in psychology. Women accounted for 80% of UK undergraduate acceptances onto psychology courses in 2019 [5], a gender differential which may be increasing and which has been observed in other countries [2,6]. In the US, the percentage of psychology PhDs awarded to men has fallen from 70% in 1975 to 30% in 2008 [7]. This is accounted for both by an increase in women graduating from PhD programmes, but also a decline in the number of men graduating from these programmes [8]. This international trend translates into the workforce, where approximately 80% of psychotherapists, clinical and counselling psychologists in the UK are women [5,9] and around 67% of psychologists in the US are women [10]. It is important to note that although women are underrepresented in senior positions in psychology [the ‘leaky pipeline’; [11,5]), there is an underrepresentation of men at lower levels.

The current gender imbalance limits the diversity of psychology as a field and as a profession. If men are not embedded across the psychological workforce, the profession does not reflect the population it needs to serve. This may translate into the gendered development of services, reflected in recent debate over whether current professional psychology referral protocols and interventions are more suited to women [12]. Some argue that if psychological services are not tailored for men’s needs and if men are not visible within the workforce, men may be deterred from seeking psychological support [13,14]). Although the impact of the under-representation of men in the discipline is unclear, professional bodies such as the British Psychological Society (BPS) have expressed concern and have called for evidence supporting a better understanding of psychology’s demographic profile [15].

There has been little examination of what may influence the decision-making processes of men to pursue (or not pursue) a career in psychology. This dearth of research is striking, given that the gender differential exists in a discipline which, in many countries, boasts large and rapidly growing undergraduate degree cohorts [14,16,17]). One suggestion is that stagnant salaries for practicing psychologists may disproportionately keep men from pursuing this career. An APA workforce taskforce in 1996 suggested that there is little evidence that women’s increased participation in psychology eroded psychology’s status, but rather that as salaries within the discipline became stagnant and the field lost prestige, men decided to pursue other careers and women filled the gap [7]. Previous research has explored the characteristics of psychology undergraduate students, highlighting gender differences in self-efficacy and expectations for valued outcomes [18], while also noting gender similarities in motivations for studying psychology [19]. A 2007 study found that students in psychology majors (both male and female) at US universities were less likely than other students to be influenced by job concerns, salary, family members or past experiences in their motivations to study psychology [20]. A more recent US study [21] found males’ and females’ reasons for choosing psychology as a major were strongly oriented to empathy and ‘helping’, including a desire to improve society, increase mental health, and curiosity about others’ behaviour.

There is also recent research from North America which indicates that the public view psychology as a feminine discipline and profession [22], and a focus group study with UK adolescents reached similar conclusions with the perception that psychology was about “*feelings and stuff*” [23]. This may indicate a limited perception of psychology as relating to particular areas of practice, such as clinical or counselling psychology, and indeed limited aspects of those roles at that. A Canadian qualitative study of male psychology undergraduates found that these students were keen to stress that gender ‘did not matter’, but at the same time, they subtly acknowledged the gender gap in the subject and explained how their psychology degree could prepare them for a job in a male-dominated field [24]. It has been suggested that those men who do elect to study psychology are not demotivated by its feminine stereotype because they value the cognitive skills they expect to gain from their psychology degree as much as their female peers [25]. Another reason that men who do elect to study psychology are not demotivated by its feminine stereotype may be that they are, in certain respects, more similar to their other-gendered peers: that the personality traits of the minority gender in a highly gender-segregated field have a tendency to differ from the population norm in the direction of the majority gender [25]. Indeed, this claim has been supported by Marrs et al. [20], who found that male psychology students scored significantly higher on the trait of openness to experience than males in non-psychology undergraduate programmes. Lastly, given that science subjects tend to be preferred by males [26], a further barrier to males’ uptake of psychology degrees may be an adolescent perception of psychology as a ‘sort of science’ [23]. Together, these reasons for the gender difference in the study of psychology at university level could be explained by Eccles’ theory [27] that gender differences in the two elements of its subjective task value (attainment value and perceived cost) are mediators of gender differences in occupational and educational choices. Eccles argues that gender-role stereotyped socialization can lead males and females to have different hierarchies of core personal values and long-range goals, and that if gender (and success in that gender role) is a core component of a person’s identity, then tasks (e.g., choosing a subject for a degree) that fulfil this role will have high subjective task value. Similarly, Social Cognitive Career Theory [SCCT; 28] proposes that effort towards a career goal includes elements of interest, self-efficacy, and expectations of certain valued outcomes; this helps to explain Sanders et al.’s [18] findings that male psychology undergraduates were less motivated/interested than females, but had higher self-efficacy and anticipated better performance (outcomes).

Although it is of value to study what influences men who already study psychology at university level, a more upstream focus on post-primary education settings is necessary to understand why young men choose not to study psychology, as well as why they do. Currently in the UK, psychology is offered in the form of optional, stand-alone qualifications in post-primary schools: as a General Certificate of Secondary Education (GCSE), which students usually take at age 15–16; and as Advanced Subsidiary (AS) and Advanced (A) Levels, which students usually complete in schools or colleges as part of their post-compulsory education (i.e., post-16 years old). Most universities will outline A Level entry requirements (grade minimums and subject requirements) for their undergraduate courses. While not all schools and colleges offer such psychology qualifications, the popularity of psychology is increasing in schools – for example, between 2020 and 2021, there was an 8% rise in entries for psychology A Level [29]. The gender gap in psychology is clearly evident by the time students study at A Level; it is the most popular A Level subject for female students, but the 8^th^ most popular subject for male students, and in 2021, 74% of psychology A Level entries were from women, and 26% were men [30]. Some recent studies (using samples that included both men and women) have attempted to identify the factors that influence students’ decisions to choose psychology at post-primary school. Students in England and Wales reported being strongly influenced by interest in the subject when choosing to study it at AS level, but prior and expected grades were very influential in their choice of whether to continue psychology to A Level; AS Level psychology was therefore seen as a low-risk option, but more was at stake when choosing the subject at A Level [31]. Cross-cultural research comparing Slovakian and English students’ motivations to study psychology at school found that common motivators were an interest in the subject and seeing the subject as an opportunity for self-understanding and interpersonal problem-solving [32].

Given that the gender difference in psychology emerges in the years before university subject choices are made, it is particularly important to consult with those who are still in the pre-tertiary/post-primary stage of education to understand their decision-making process. Studying pre-tertiary students’ motivations to study psychology is also important because it may alert university faculty to the needs and expectations of their incoming students [32]. Qualitative research can provide a rich exploration of the mechanisms informing this decision. With the exception of Mercer and colleague’s [23] study which was conducted a decade previously, there is no research to address this important gap. The aim of the current study is to develop an understanding of the perceptions and motivations of adolescent and young adult men in a UK post-primary education setting regarding studying psychology at university undergraduate level.

### Research questions

i. What are the perceptions of adolescent and young adult men in post-primary settings of the study of psychology at A-level and undergraduate level?
ii. What are the perceptions of adolescent and young adult men in post-primary settings of the career prospects for psychology graduates?

## Method

### Study design

A focus group study utilising reflexive thematic analysis [RTA; 33] was employed. A focus group method was chosen to enable understanding of a wide range of experiences, perceptions and attitudes, with additional data from group interaction. In this study, reflexive thematic analysis is employed from a critical realist ontological perspective to provide an interpreted reflection of reality. As a methodology this fits our interest in prioritising adolescent and young adult (AYAs) lived experience and subjective understandings of the study of psychology, since it is underpinned by the cultural and social resources of both participants and researchers [34,35]. We acknowledge that there is an external reality independent of our perceptions, yet believe this is shaped by the perspectives and contexts of both the participants and researchers. This design and methods of this study are reported according to the RTARG guidelines for thematic analysis [36].

### Setting

This study was undertaken in collaboration with post-primary schools in Northern Ireland (UK). Psychology is offered as an A-Level in some, but not all schools, and uptake of the subject is low in comparison to other subjects - psychology represented only 1.7% and 1.8% of the total of AS and A2-Levels taken in 2022 [37]. Out of the 191 post-primary school settings in NI, the majority are co-educational (80%) with 9% boys-only, and 11% girls only [38].

### Identification and recruitment of participants

Purposive sampling was undertaken within participating schools to identify and recruit AYAs aged 16 years of age or over, who identify as a man, and who were able to provide informed consent. AYAs were stratified into the following three groups: i) studying psychology (at AS or A2-Level), ii) not studying psychology, but studying at least one other STEM subject (at AS or A2-Level), and iii) not studying psychology nor any other STEM subjects (at AS or A2-Level). In each school, the following number of focus groups participated: School 1: four psychology focus groups (FG1, FG2, FG3, FG4); School 2: one STEM and one non-STEM (FG5, FG6), School 3: three psychology focus groups (FG7, FG8, FG9), School 4: one non-STEM and one STEM (FG10, FG11), and School 5: one individual STEM interview (I12).

Prospective schools were contacted and provided with information on the study, with permission sought from the school principal. A total of five schools agreed to participate in the research. Of these, two offered psychology as an A-Level subject (School 1 and School 3). One school was co-educational (School 1); the remaining four were boys-only. All participating schools were voluntary grammar schools. In one school the proportion of pupils receiving free school meals (an indicator of deprivation) was above the average for NI, indicating higher deprivation (School 4).

Although saturation is not recommended with reflexive thematic analysis, information power can inform sample size estimation [39]. At least two focus groups per stratum are recommended to provide a more comprehensive understanding of issues in order to fully capture nuances of conceptual codes [40]. With three strata, desired sample size for the current study was 6–12 focus groups (of 6–8 participants each) and thus an overall sample of 48–96 participants. A total of 64 participants were included in the 11 focus groups and 1 single interview, with between 4 and 8 participants per focus group. This was in line with our need to capture a range of perspectives, with the final sample size shaped by the adequacy (richness, complexity) of the data for addressing the research questions [40]. Specifically, out of 64 participants, the sample consisted of 1) 36 participants studying Psychology (of which 15 were studying Psychology and at least another STEM subject, 21 were studying Psychology and other non-STEM subjects); 2) 24 participants not studying Psychology (of which 16 were studying STEM subjects and 11 were studying non-STEM subjects).

Principals of participating schools identified a teacher to distribute participant information sheets (which included the study aim) and consent forms to eligible AYAs. If interested in participating, AYAs signed the consent form and returned this to the designated teacher. Data were collected between 5^th^ April and 31^st^ May 2022. The majority of students were aged 17 (46.9%) or 18 (45.3%), were part of a larger family of at least 2 siblings and had a mother educated to at least degree level (see Table 1).

**Table 1:** Socio-demographic characteristics of focus group participants (n = 64).

	Frequency	%
**Age**		
16	5	7.8%
17	30	46.9%
18	29	45.3%
**Number of Siblings**		
0	4	6.3%
1	15	23.4%
2	25	39.1%
3	13	20.3%
4	5	7.8%
5	1	1.6%
10	1	1.6%
**Mother’s Highest Level of Education**		
No formal qualifications	3	4.7%
GCSEs, Junior Certificate, or equivalent	12	18.8%
A levels, Leaving Certificate, or equivalent	9	14.1%
Higher National Certificate or Higher Certificate	3	4.7%
Bachelor’s Degree or equivalent	15	23.4%
Master’s Degree, Postgraduate Certificate/ Diploma, or equivalent	13	20.3%
PhD or equivalent	3	4.7%
I would rather not say	6	9.4%
**Studying Science or Maths**		
Yes	25	39.1%
No	39	60.9%

### Focus groups

Suitable dates/times were agreed to conduct focus groups in quiet rooms at each school. A focus group schedule (Supplementary Material 1) was developed based on the aims and objectives of the study and a review of the research literature. The focus group guide included open-ended questions asking participants to describe their experience of, perceptions of, and attitudes towards the study of psychology. The schedule was piloted prior to data collection, prompting minor changes to reduce its length. Participants were also asked to provide socio-demographic details.

Focus groups were facilitated by one or more experienced researchers (*KG, RL, SB, EC*), all women lecturers/postdoctoral researchers with PhDs, with a second moderator taking notes (*LGW, TA, GD, EC*). None of the research team had prior knowledge of the participants. Focus groups lasted between 15 minutes to 60 minutes and were audio-recorded on an encrypted digital transcription device and transcribed verbatim. All facilitators held faculty member positions in the field of Psychology, and while their affiliation with academia facilitated access to schools and the organisation of focus groups, it also rendered them insiders in this research from the perspective of the discipline of interest. Two authors acted as student researchers for the initial warm-up sessions due to their closer proximity in age to the participants. However, the female in-school research team arguably remained positioned as outsiders from the participant perspective as the A-Level students were significantly younger and male. We remained aware of the need to navigate power dynamics and to take action to mitigate influencing students’ perceptions of psychology. We explicitly acknowledged our insider-outsider positionality with participants during the focus groups; we assured participants of confidentiality, that there were no right or wrong answers; and we informed them that their names would not be revealed in published work. After taking part in focus groups, participants were provided with a printed debrief (including career advice provided by the British Psychological Society), and were offered a small gift in recognition of their time (a pen or t-shirt).

### Analysis

The analysis of the data was supported using NVIVO software and was informed by reflexive thematic analysis [39]. Using an inductive approach to data analysis, three experienced researchers and authors of this paper first familiarsed themselves with the data by reading and re-reading the interview transcripts. The data was then sorted into nodes in NVIVO, where key features of the data relevant to the research questions were identified. Afterwards, the researchers engaged in collaborative discussions to refine initial codes and begin the process of theme development. This step involved generating initial themes by grouping together related codes, reflecting on patterns and meanings across the data.

As the relatively large, stratified sample and broad interview protocol allowed insights into both the depth and breadth of students’ perceptions of psychology, we thoroughly reviewed the data multiple times, aiming to capture the nuanced experiences of the student groups. The process of theme construction was iterative and reflective, meaning it was continuously refined over time through regular discussions between the three analysts. This allowed for ongoing validation of ideas and exploration of assumptions about and interpretations of the data.

As one of the research goals was to explore differences in motivations between students who study A-Level psychology and those who do not, a constant comparison approach [41] was used which prioritised the analysis of data from these distinct groups. This comparison was crucial to ensure that the final themes reflected the distinct perspectives and experiences of these groups.

### Ethics approval

Research ethics approval was provided by the Queen’s University Belfast Engineering and Physical Sciences Faculty Research Ethics Committee (Reference; EPS 22_22) on 4th February 2022. The study was conducted in accordance with the Declaration of Helsinki and participants completed an informed consent statement prior to completion of the focus group. Safeguarding children’s policies and COVID-19 risk assessments were adhered to.

## Results

**Fig 1:**
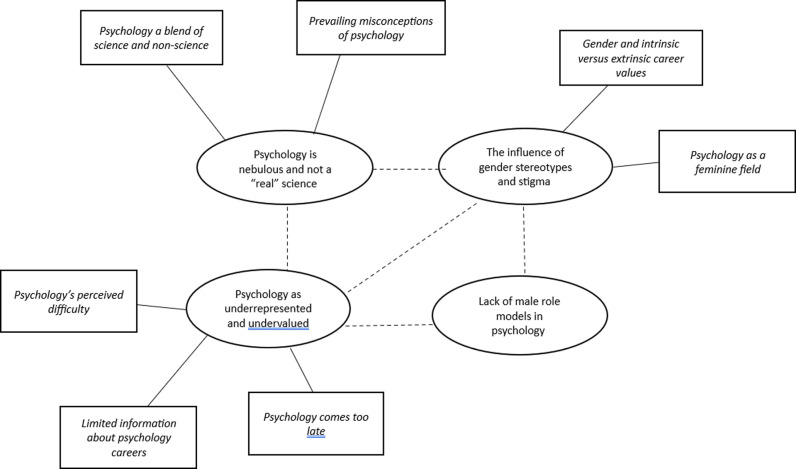
Thematic map of themes and subthemes.

We constructed four themes reflecting findings on motivations that encouraged or discouraged young people to study A-Level psychology: (1) Psychology is nebulous and not a “real” science; (2) Psychology as underrepresented and undervalued; (3) The influence of gender stereotypes and stigma; (4) Lack of male role models in psychology (see thematic map below, in Fig 1).

### Theme 1. Psychology is nebulous and not a “real” science

Generally, the predominant discussion around young men’s perceptions of psychology was centred on the complexities of defining and positioning the discipline. Across all groups, participants expressed a lack of knowledge and numerous misconceptions about psychology as a subject, revolving around whether psychology is considered a science, the content of the field, and the opportunities that it offers to students.

### Psychology as a blend of science and non-science

Participants had mixed views about how they perceived psychology to be a science or non-science subject. Both STEM and non-STEM students held a shared belief that psychology is not exclusively a STEM subject. This perception arose from the belief that psychology diverges from the objective and ‘fact-based’ nature of other STEM subjects. Psychology, in contrast, was considered to be more subjective and ‘harder to verify’. Although proportionately, more psychology A-Level students overtly described psychology as a STEM subject than did groups from other strata, some of them inadvertently discussed limitations of psychology as a whole studying behaviour in a scientific way: “It *[psychology] just varies loads. Like there’s completely different topics like. Like some topics can be about understanding someone’s behaviour and then completely science in the next topic’* (P3, FG9, Psychology). Their views pointed towards psychology as a blend of both science and non-science, attributing this view to the subject’s diverse range of ‘social’ topics and research methods. Therefore, they situated psychology within STEM and social science disciplines. They justified this perspective by referencing applications of psychology, such as understanding and helping people, which they felt contrasted with applications of what they considered canonical STEM subjects.

*In psychology there’s, it’s just fluid whereas other ones are set in stone, like math is absolute* (P1, FG6. Non-STEM)

*It’s kind of like a middle ground, I guess, ‘cause there is obviously scientific method within it, but it’s not entirely a science. It could also be like a social science in that aspect of just how people work, rather than the science of, in terms of chemicals or whatever, so it is kind of just a middle ground between that. It doesn’t have to be one or the other.* (P3, FG6, Psychology)

These quotations illustrate how psychology is seen as straddling both scientific and humanistic paradigms.

***Prevailing misconceptions of psychology.*** Participants tended to seek clarification from the interviewer on whether Psychology was formally considered a STEM subject and were often surprised that it was classed as such in the UK. Students studying psychology at A-Level reported misunderstandings of others about the discipline as largely focusing on ‘emotions’ and ‘helping people’ and also of what it enabled students to do. For that reason, many perceived psychology as akin to sociology, as both fields were understood to examine how society operates and how individuals within society function.

*I just think when I hear it right away, the social side of it and helping people and like trying to understand people. […] That’s like what I think when I think of psychology.* (P4, FG11, STEM)

*I think a lot of people get the wrong perception of it. All of this, thinking it’s all about the mind when it’s actually understanding different disorders and like it’s not just how the brain works. […] It’s more diverse than what they think. Like it’s not all brain.* (P7, FG9, Psychology)

These quotes collectively demonstrate the complexity of defining psychology and the breadth of the field, emphasising its diverse applications and common misconceptions that surround it.

### Theme 2. Psychology as underrepresented and undervalued

Several barriers were discussed relating to how psychology is resourced and positioned within post-primary and tertiary education. Young men perceived themselves to have less exposure to the subject at an earlier stage, which influenced the perception of psychology as a unique and disconnected subject to study. This was coupled with what participants perceived as a lack of high-quality information about careers in psychology, both from school and universities.

***Psychology comes too late.*** At GCSE level, psychology is perceived as predominantly offered in all-girls schools (e.g., by the examination board AQA), which was seen by participants as providing valuable early exposure to the field and its potential career prospects. Non-psychology students discussed the lack of availability of GCSE psychology as an option within their schools, with several participants from both STEM and non-STEM groups stating that if they had the option of studying it at GCSE level, they would have chosen it. To choose an unfamiliar subject to study at A-Level was seen as ‘a risk’.

*I would have definitely chosen it two years ago if I would have been exposed to it.* (P1, FG11, STEM)

*P3: I feel like if it was studied at GCSE there would be a whole lot more people doing it at A and AS level. […]*

*P1: I think that there’s a lot of uncertainty about going into a new subject - people usually just stick to what they did at GCSE rather than broaden their range. I think that just adds to it really yeah.* (FG2, Psychology)

The above excerpts illustrate that information about psychology was often perceived as arriving too late, implying that students have already formed intentions about their preferred areas of study. This lack of exposure and underrepresentation of the field seem to contribute to the prevailing unfamiliarity surrounding psychology, influencing how seriously it is taken by students at A-Level.

*Yeah. Like the fact that I’m only finding out now that it’s a STEM subject, […] shows that has, like it’s very underrepresented even within school* (P1, FG6, Non-STEM)

*I feel if it was presented better, it would be viewed as a more serious subject which it is.* (P8, FG2, Psychology)

Students in non-STEM groups in particular had a perception that “essay-based” subjects (a description that they believed to apply to psychology) were ‘downgraded’ and ‘overlooked’ at school, and that more emphasis and resources were placed on maths and other STEM subjects.

***Perceived difficulty of psychology.*** Unfamiliarity with psychology as a subject supported an initial perception of a ‘soft’ and ‘easy’ option at A-Level, and an unexpected challenge to those opting to study it. There was the perception that those not studying psychology viewed it as an easy subject:

*I think some people would view it as an easier A-Level or something like that as I think most people would think, like generally when they think of A-Levels they would think of the big four that are extremely difficult, like further maths or I suppose some people would say chemistry.* (P1, FG12, STEM)

Some psychology students described perceptions of the subject from those who did not study it as being more similar to arts-based or vocational qualifications. They also noted that any indication that a subject was enjoyable rather than purely mentally taxing suggested to others that it was ‘soft’.

*Like we’ll get up and eh you know like be like “we’re away to psychology” but that’s with the same as any subject that isn’t STEM, I think if you said you’re going to media studies your mates would have a go at you […].Because like if it’s a subject we enjoy I think they see it as like, unless you’re like completely bogged down in work and you don’t understand anything then it’s considered a, a soft subject. […] But they’d realise if they did it like it’s not a soft subject.* (P1, FG1, Psychology)

A-Level psychology students themselves perceive it as a difficult subject, finding the content and learning approach unfamiliar and disconnected from other subjects (and therefore especially challenging). Aspects of the subject which psychology students perceived as overly complex and technical included research methods and statistics and understanding theory and terminology. “*Sometimes the theories are quite hard to wrap your head around, you have to read them quite a lot after as well*.” (P1, FG2, Psychology).

Moreover, A-Level psychology students viewed themselves as being disadvantaged, as the subject was perceived as requiring mastery of large amounts of diverse information. Required learning outcomes were reported to be broader and therefore more demanding than those of other subjects. In addition, psychology was also seen to require a specific approach to taking exams, including time management techniques, essay writing skills, and understanding how to answer questions without a ‘*set answer’* (P2, FG2, non-STEM).

***Limited information about psychology careers.*** In addition to limited information about psychology as a subject, participants, particularly those in schools without psychology offerings, believed there was a lack of information about potential career options related to psychology. Many A-Level psychology students chose the subject because they found it interesting, yet lacked knowledge about professions requiring psychology or other fields where skills gained from studying psychology could be beneficial: “*I don’t know many jobs that require psychology; I’m sure there’s a plethora out there, but we’re not told about any, really*.” (P3, FG1, Psychology).

While career options associated with other subjects (particularly STEM) were promoted through posters and classroom materials, as well as more informally through conversations, participants felt that psychology did not receive the same attention and to study psychology at A-Level was regarded as an unusual choice. Participants noted that the information provided by the Universities and Colleges Admissions Service (UCAS) in the UK about psychology at university level was insufficient, lacking details about the course content and potential careers after earning a psychology degree. Consequently, many students opted for psychology A-Level due to its broad nature, offering flexibility for diverse career paths rather than a specific focus on psychology-related professions.

*And just not having enough information about it would tend to be like ‘Oh, I might choose this because it gives you a broader, uh, career path and experience you can go towards’* (P2, FG10, non-STEM)

Participants suggested that universities should provide a clearer classification of psychology as a discipline. There was a consensus that explicit categorisation as a science could enhance its appeal, potentially viewed as a pathway to prestigious fields like medicine. Conversely, if psychology was classified as a social science, concerns were raised about its perceived limitations in supporting desirable careers. Some participants emphasised the need for targeted guidance, especially for young men, regarding potential careers where psychology could be beneficial.

*I feel like we could have benefited a lot actually if they came in and told us like maybe not just like, not jobs that exclusively require psychology but in subjects where it would help and like fields where it would make sense to do psychology* (P3, FG1, Psychology)

In summary, there appears to be an opportunity in terms of increasing awareness of the breath and transferability of psychology as a discipline of study.

### Theme 3: The influence of gender stereotypes and stigma

Findings suggest that prevailing gender stereotypes significantly shape young men’s motivation to study psychology. STEM students, influenced by these stereotypes, perceived psychology as a feminine discipline, assigning it lower value compared to other fields.

***Psychology as a feminine field.*** The prevailing societal perceptions of psychology seem to adversely impact the choices of young men regarding the field as a future area of study and career. Psychology is perceived as a feminine field, and choosing it was seen as a potential threat to men’s masculine identity, with the risk of ridicule and social judgment “*Yet there’ll still be maybe, some men might feel de-masculinised doing a subject that we perceive as feminine*.” (P2, FG5, STEM). Men who chose psychology were seen as risking ridicule, judgement from other men, and one participant even described it jokingly as “*social suicide*” (P1, FG11, STEM). One participant noted the “*courage*” required for men to step outside gender norms (P4, FG11, STEM).

*And building on that point earlier about, eh, toxic masculinity and all, you’d have to question if people would see a man with a psychology degree and see it as a real degree for him if you get me, and those like ‘What are you doing that for?” and “That’s not a man’s degree”. And see that as like a deal breaker with him almost.* (P4, FG6, non-STEM)

Participants reported being pushed towards subjects that were perceived as stereotypically masculine from a young age (‘hard’ science subjects such as engineering or maths): “*men are much more pushed to STEM and I know Psychology is STEM, but it isn’t accepted as such”* (P4, FG6, Non-Stem). These stronger traditional views about which fields are exclusively STEM and more suitable to men were held mostly by STEM students not taking psychology as an A-Level subject.

These social views more broadly shared by all students appear to be reinforced by the perception that psychology primarily focuses on emotions, which were thought to be less frequently discussed by men due to the pressure of conforming to their gender stereotype. As men tend to disassociate themselves with fields that would jeopardise their traditional gender views this influence makes young men hesitant to pursue psychology in the future.

*I think that sort of puts men off, where they feel like scared or something that they have to talk about their feelings. […] And if you try and tell people that, aw you’re studying psychology, they’d sort of look at you a bit differently. Just being like, that’s a bit of a weird thing to do, just ‘cause you’re a boy and you’re not really meant to show your emotions.* (P4, FG11, STEM)

When asked about others’ perceptions about psychology, participants across different groups cited perceived evolutionary differences between men and women as reasons for men being less suited to psychology careers. This perception included beliefs that men are less ‘caring’ and ‘trustworthy’ than women. The stereotypes extend to viewing psychology as a predominantly feminine career due to assumptions of women’s greater resilience and ability to maintain a work-life balance in an emotionally demanding career. Some participants in a non-STEM group argued that girls mature earlier, make better decisions, and achieve academically, enabling them to pursue psychology which was perceived as a broad and internally fragmented subject “*I think it’s also our maturity levels… […] Like it’s a known fact, whether society that makes women mature faster or if it’s just genetics, but women do mature faster* (P5, FG6, non-STEM).” These findings highlight how gender stereotypes not only frame psychology as a feminised field but also reinforce beliefs about men’s unsuitability for the profession

***Gender and intrinsic versus extrinsic career values.*** In addition to the gendered conceptions and societal pressures surrounding suitable careers for men and women, career aspirations were perceived to be influenced by distinct motivational factors for each gender. Women were described as having more intrinsic interest in ‘helping people’ and in ‘caring’ professions, with aspirations for a positive impact on society. In contrast, men were thought to be primarily motivated by extrinsic values of high financial rewards, which were seen as more attainable through traditional STEM careers, rather than being driven by altruism or by improving others’ lives, characteristics associated with women’s intrinsic interests.

*It’s like wrong to generalise but in general I think women would be more empathetic and more, more willing and ready to want to help. […] Whereas men are more sort of, self-centred in a sense. Or they’d get a job where they focus on themselves instead of thinking, “oh maybe, maybe I could help people with this and have a good job and I’m making money”.* (P1, FG10, non-STEM)

Participants suggested that these motivations develop early in life. The prospect of postgraduate study and the additional steps required to become a psychologist were therefore viewed as discouraging, delaying financial and promotion prospects: “*I want to make money as soon as I’m done*” (P4, FG6, non-STEM) and “*as a man, you pick your career path straight away to get to a certain level*” (P2, FG10, non-STEM). The perception of psychology as a female-dominated profession led to concerns about limited opportunities, and some men considered the perceived status of psychology in the wider community as a potential drawback: “*it’s not like someone saying ‘I’m gonna be a lawyer or a businessperson… I’m gonna be a psychologist, who says that?!* [laughs and others laugh too]’ (P5, FG10, non-STEM). These findings highlight the persistent misconceptions about what a career in psychology entails, reinforcing that it offers solely intrinsic rewards—an association that may deter those who prioritise financial gain.

### Theme 4: Lack of male role models in psychology

In terms of the influences on their A-Level and university subject choices, participants listed a range of people, most commonly mentioning family and people they knew who had studied a particular subject. Some described information provided by schools as being an influence on their subject choices, for example, teachers motivating students to study the subject “*Probably school like teachers saying you are well in this subject you can do well in A-level*”. (P2, FG5, STEM).

Of those with exposure in their social circle to someone who had studied psychology at university level, experiences were largely positive.

*My uncle has studied psychology and he’s em, he works as a clinical psychologist, so I’d say that has influenced me quite a lot. […] I would say he’s always giving off a very positive image of the subject and he has always made it seem very interesting. I think it’s always been one of those jobs to me that seems like it’s very rewarding.* (P1, FG12, STEM)

The lack of male role models and peers in psychology was perceived across participants to be a barrier for young men pursuing the study of psychology. Participants noted the female dominance in A-Level psychology classes. Psychology A-Level students in the co-educational school reported greater numbers of females in classes than males, with only one group reporting a 50/50 split class. The observation of more female psychology teachers further shaped the young men’s decisions about whether to study psychology at A-Level, with students suggesting an increase in male teachers could attract more male students: “*I feel like if there were more male teachers more males would choose to do it*.” (P3, FG2, Psychology).

Media and peer influence reinforced gender stereotypes, portraying psychology as a subject where men must challenge norms and face potential judgment.

*It’s just, and even if you look on websites and stuff, it would be like, like two girls holding books for psychology and then like you look at computer science or film, or whatever, and it’d be like men or women. […] I feel like if you’re looking at that, you’re like “aw I don’t really want to do that” in case you’d get like judged for being, doing psychology because it’s seen as more of a girly subject than like a male subject.* (P2, FG8, Psychology)

Despite this, some participants viewed men’s under-representation as an opportunity. They chose psychology for A-Level based on career advice, anticipating high value to potential employers and university admissions.

## Discussion

The current study is one of the first to examine the perceptions of young men in post-primary education towards the study of psychology in higher education. Mercer and colleagues [23] contributed the only other qualitative study on this topic which the authors are aware of, conducted 10 years previously and also in the UK. Currently in the UK, one in 20 university undergraduate students study psychology; admissions have doubled since 2013 [42] and the gender differential in intake has also arguably widened [2]. The current study suggests that this may have occurred in a context of prevailing gendered perceptions of psychology, a subject which is viewed by young men as ‘feminine’ and ‘soft’, and as largely centring on feelings and emotions. To study psychology as a young man is perceived as non-conformist and potentially threatening to one’s masculine identity. Psychology is also still perceived as being positioned between a ‘real’ science and a social science, with young men struggling to define the scientific endeavour as it applies to psychology. The current study goes further in advancing our previous understanding by elaborating on the mechanisms behind these prevailing perspectives.

Young men in the current study found it challenging to define psychology as a discipline and as a science. A simultaneously nebulous yet narrow view of psychology as largely focusing on ‘emotion’ and ‘helping people’ was presented, even by those studying the subject at A-Level. Interestingly, an emphasis on helping others appeared to differentiate psychology from other STEM subjects. This raises questions about student perceptions of wider STEM, where a central pedagogical tenet is solving real world problems to benefit society [43]. Lilienfeld [44] notes the public’s scepticism about psychology’s scientific status can partly be attributed to a misunderstanding of the nature of psychological science. Psychology is a strongly evidence-based discipline, yet this is not the prevailing perception [45].

Further, young men in the current study found the study of statistics and theory challenging, reflecting the perceptions of English post-primary students [23], but in contrast to Slovakian students’ perceptions of psychology [32]. Men in our study also perceived choosing to study psychology at A-Level or higher level as ‘a risk’, with most having no prior exposure to the subject (reflecting the national trend for general uptake of A-Level psychology in NI reported earlier [37]. This may also be contributing towards the ‘othering’ of psychology reported here - its lack of integration with other STEM subjects in the post-primary curriculum limits the ability of students to see the various connections between key skill development in psychology and wider STEM. The consequences of psychology not enjoying clear positioning within STEM may be significant, with the young men in the current study discussing an indirect gendered impact from a devaluing of the discipline by teachers, career staff and the wider school system.

The young men in the current study felt that psychology had not been actively promoted to them within post-primary education as a career option, either by teachers or careers staff. This could suggest that teachers demonstrate an implicit gender bias [46] which leads to psychology being promoted more to young women. The participants in the current study felt that traditional STEM subjects were promoted more in their schools and that a stronger positioning of psychology within STEM would make the subject more attractive to young men due to its potential to lead to more prestigious careers, appealing to extrinsic values. Changes in the marketplace and stagnation of salaries within the psychology profession have previously been suggested as a reason for the gender differential [7]. This relates to societal pressures on men as ‘breadwinners’ and may support Social Cognitive Career Theory [47] as a useful framework to explain gender differences in expectations of valued outcomes from pursuing a career in psychology.

The perceptions of young men in the current study add to the evidence base that psychology is a field of study predominantly associated with femininity [22,23], and that these beliefs are ingrained by adolescence. Mercer and colleagues [23] suggest that as psychology is a multi-faceted subject incorporating a number of strands, topics may be seen as differing in their gendered associations. The young men in the current study largely perceived psychology in terms of topics with significant gender associations (i.e., the study of emotion and feelings), and implicitly had a narrow perception of psychology as encompassing clinical psychologists supporting the general population’s mental health. The young men discussed how studying psychology was potentially threatening to one’s masculine identity, supporting Eccles’ [27] claim about the importance of the centrality of gender identity to an individual in relation to their educational and occupational choices. These findings have similarities to findings about public perceptions of the nursing profession [48]. Psychology may similarly be defined as ‘a science applied to caring’ in relation to those who practice, though it appears to align more to nursing in this respect than medicine which does not have the same gendered associations. It has been suggested that the personality traits of the minority gender in a highly gender-segregated field have a tendency to differ from the population norm in the direction of the majority gender [25]. There was frequent evidence of implicit gender bias in the current study, with discussion of personality differences between genders as a motivating factor for studying and achieving within psychology. Indeed, there is some evidence that highly empathic students may choose psychology because they believe that empathy is important for success in clinical and counselling psychology [6,16]. This can potentially be explained by the Theory of Precluded Interest [49], which proposes that societal stereotypes can deter individuals, particularly those from underrepresented groups, from pursuing interests or careers in specific fields due to a perceived incongruence with their identity. Relating this to psychology, the current study identified a perceived lack of men in psychology as role models. This could contribute towards young men not seeing themselves as similar to idealised group members, both in relation to their gender and perceived gender-specific traits.

The current study evidences several key differences in the perceptions of psychology between young men who study psychology at A-Level, and those who do not. Firstly, A-Level psychology students were more likely to identify psychology as a science and as a broad and engaging subject. They also perceived psychology to be challenging, unlike their non-psychology peers, particularly in relation to components relating to research methods, statistics and theory. These components are core within the undergraduate psychology degree programme in the UK and internationally [50]. Together, these findings suggest that exposure to the subject in pre-tertiary education may facilitate a more accurate perception of the study of psychology in higher education, potentially facilitating applications but also reducing student attrition. In practical terms, it may be of value for higher education institutes to develop targeted partnerships with pre-tertiary education providers to provide careers workshops and engagement activities where there is no existing subject-level provision. Lastly, A-Level psychology students appeared to be aware of, yet not hold, the gender stereotypes about the subject which were expressed by their peers. This might suggest that even in pre-tertiary education, young men opting to study psychology may be those who are less influenced by external pressures, like their counterparts in higher education [20]. Alternatively, with the current study evidencing a potential link between gendered associations and inaccurate perceptions of psychology as a subject, it may be that the study of psychology helps to diminish pre-existing gendered associations.

The current study has a number of strengths. Primarily, we have addressed a call of evidence from several professional bodies internationally to help explain the gender differential in the psychology workforce. We provide the most robust qualitative study of young men’s perceptions of psychology in post-primary education settings to date, with a large sample which is reflective of the student population in their study of STEM and psychology at A-Level. The current study implicates young men’s perceptions of psychology as a highly gendered field and ‘not a real science’ as mechanisms supporting the gender disparity in the psychology workforce, and we align these to several psychological theories. This provides a firm foundation for future research. There are also a number of limitations. First, the data collection was focused on one UK region, and the psychology GCSE and A Level provision and curriculum varies in geographical terms. The current study was also limited in respect to a focus on (largely boys-only) secondary schools and did not sample students from further education colleges who may better reflect the diversity of students going on to study psychology at higher education level. Additional socio-demographic information (e.g., ethnicity) would have been useful to assess the representativeness of the sample. Lastly, the use of focus groups may have resulted in social conformity within the discussion; however, we believe the richness of data precludes this impact.

This is a significantly underdeveloped area of research and is in sharp contrast to large bodies of existing research to explain gender differentials in other disciplines, such as nursing. There are therefore various fruitful avenues for future study. The current study has demonstrated that gendered perceptions of psychology exist in young men between 16 and 18 years of age. This would suggest that early intervention is needed, although more research is needed with younger adolescents and children to understand how and when such perceptions of psychology develop. The current study is focused on one UK region, with previous research in this area also conducted in high-income countries. Given the ‘gender-equality paradox’ where gender segregation is more pronounced in developed countries [51], research in low-and-middle-income countries would be valuable to explore if the same implicit gender biases exist. Findings in the current study suggest a lack of support for pursuing psychology as a career within post-primary settings. This needs to be investigated in more depth, and research to understand the perspectives of both subject teachers and school career advisors would be valuable. Similar qualitative endeavours with young men already in higher education who chose to study psychology may help to further understand some of the identified mechanisms. This research could also be extended to trainees on professional doctorates, where a gender differential is also evident. Lastly, large-scale, theoretically-informed quantitative work is needed to model the processes and outcomes of young men who choose to study psychology at school and university, so we gain an understanding of both recruitment and also retention of men. Our research group is currently working to address this.

There are various applications from the current study findings for public engagement, the post-primary education setting and the higher education setting. If the prevailing perception of psychology of the male adolescent public at large is congruent with those of our participants, there is a need to enhance the way in which we promote psychology to the public. This is both in relation to psychology as a science and psychology as a multi-faceted discipline. There is an acknowledgement of psychology as the study of human behaviour, but little understanding in terms of the breadth of that term as commonly employed in psychology. In the post-primary education setting, this could be supported with greater provision of psychology as a subject at an earlier age (GCSE) and clearer positioning and promotion within STEM. Through this endeavour there is opportunity to enhance student understanding of what science is more broadly, and to engage in debate on the nature of science. Professional bodies such as the BPS and regulatory bodies could work together to ensure psychology as a scientific endeavour is a central tenet of post-primary curriculums. There was also frequent evidence of implicit gender bias throughout the focus groups, and greater attention to the psychology of gender within the post-primary psychology and/or sociology curricula may help modify these beliefs. This could help influence young men not to see themselves as an outgroup in relation to pursuing psychology as a career and may have broader societal benefit.

Higher education organisations can help support post-primary education through more targeted outreach with young men. In the current study, there was suggestion that universities could more explicitly define psychology as a science in recruitment materials and monitor the dominance of women in images. A co-design approach to developing careers resources for schools may be valuable, working closely with young men, psychology teachers and careers staff to ensure recruitment materials clearly position psychology within STEM and do not reinforce gendered associations. Promotion of men in psychology is also key, in terms of contact with adolescents and young men to address the lack of role models identified in the current study. This suggests that the inclusion of male staff and students in school outreach activities is likely to be influential, particularly those working outside of fields associated with mental health or emotion. School outreach could also extend to targeted activities around mentoring, currently employed with under-represented groups at university level. Lastly, higher education organisations could help provide training and guidance to teachers and school career advisors to ensure gender inclusive career guidance is provided to all students. Greater collaborative working both between universities and with professional bodies would support resourcing these actions, given the scale of the issue.

## Conclusion

In conclusion, the current study provides evidence that young men have gendered perceptions of psychology and for the first time, suggests a gendered impact of scepticism over psychology’s place within science. The findings will inform future research in this area addressing the marked gender differential within the psychological workforce and suggest a number of actions which could be taken now with the general public, in post-primary education settings and in higher education. The global Athena SWAN network [52] which seeks to address gender inequality, is one potential vehicle to catalyse collaboration on addressing the lack of gender representation in psychology.
